# Relationship of Gallbladder Perforation and Bacteriobilia with Occurrence of Surgical Site Infections following Laparoscopic Cholecystectomy

**DOI:** 10.1155/2015/204508

**Published:** 2015-10-29

**Authors:** Nikhar Jain, Sushanto Neogi, Rajandeep Singh Bali, Niket Harsh

**Affiliations:** Department of Surgery, Maulana Azad Medical College and Lok Nayak Hospital, New Delhi 110002, India

## Abstract

*Aim*. To assess the occurrence of SSIs in patients with spillage of gallbladder contents and bacteriobilia during laparoscopic cholecystectomy. *Methods*. We evaluated 113 patients who underwent laparoscopic cholecystectomy between September 2013 and April 2015. The SSIs and their relationship with gallbladder rupture and bacteriobilia were assessed. *Results*. The mean age of patients developing SSIs was 45.57 ± 8.89 years. 18 patients (16%) had spillage of bile from the gallbladder. Percentage of SSIs overall was 6%, while percentage of SSIs in gallbladder content spillage was 5.5%. Organism profile of the culture from surgical site showed monomicrobial infection: 58% *Staphylococcus aureus*, 14% *Pseudomonas*, and 14% *E. coli*. The occurrence of SSIs in patients with bacteriobilia was 16% as compared to 2% in patients without bacteriobilia. *Conclusions*. Gallbladder content spillage is not a significant risk factor leading to increase in SSIs. The occurrence of SSIs is significantly higher in patients with bacteriobilia.

## 1. Introduction

Surgical site infections (SSIs), a significant postoperative complication, can lead to considerable patient morbidity and mortality. Preventing SSIs is an essential factor in improving the results of surgical procedures. Various factors may be associated with frequency of SSIs in laparoscopic cholecystectomies [LC] like method of disinfection of the laparoscopic instruments, microdamage to the reusable instruments, bacteriobilia and gallbladder content spillages, use of antibiotics, and so forth. The rise in demand for minimally invasive surgery has resulted in increased frequency of gallbladder perforations. Laparoscopy-attributable mortality reaches 0.5%, morbidity reaches 4%, and SSIs rates following this procedure range between 0.1 and 2% [1]. Although a number of studies have focused on the implications of gallbladder content spillage during LC, little is known about the risk factors for SSIs in LC. Here we compare the clinical outcomes in patients with gallbladder content spillage during LC and the role of bacteriobilia in SSI after LC.

## 2. Aim

The aim of this paper is to assess the occurrence of surgical site infections in patients with spillage of gallbladder contents and bacteriobilia during laparoscopic cholecystectomy.

## 3. Patients and Methods

This study was conducted in 113 patients who underwent laparoscopic cholecystectomy (LC) during a period of 20 months at the Department of Surgery, Maulana Azad Medical College and Lok Nayak Hospital, New Delhi. It was a prospective observational study. All patients more than 18 years with symptomatic cholelithiasis proven by ultrasonography were included in this study. Patients <18 or >70 years of age, having evidence of cholangitis, pancreatitis, and previous biliary tract surgeries, and having immunocompromised state or diabetes mellitus were excluded. Patients with acute cholecystitis were excluded from our study group as per the protocol in our institution to manage it conservatively, followed by interval cholecystectomy. The data were collected in all patients who underwent LC, to determine the risk factors and outcomes of gallbladder content spillage, that is, the type of content spillage, duration of surgery, pericholecystic adhesions, bile culture and antibiotic sensitivity, and wound culture if SSIs occurred.

All operations were performed under general anaesthesia. All the patients in our study received a single dose of ceftriaxone 1 gm half an hour before the infraumbilical incision was made. Standard conventional cholecystectomy was carried out in all patients by the same surgeon to prevent operator bias. If gallbladder rupture and gross spillage of bile or stones were encountered, spilled stones were retrieved in an Endopouch and local peritoneal lavage with copious amounts of saline was performed. Routinely the gallbladder was removed through the umbilical port. The decision to leave a closed system suction drain was left to the operating surgeon. A sample of bile was taken from the gallbladder and sent for bacterial culture and sensitivity in all patients.

The surgeons assessed the suitability for discharge usually on the second postoperative day. After discharge the patient was called after 1 week for proper clinical assessment of the surgical site. Thereafter patient was followed up weekly for 30 days. If local signs of inflammation or purulent discharge from the wound were present, stitches were removed and the pus was sent for culture and sensitivity. Ultrasonography for collection was done in patients with clinical suspicion of deep infection, temperature >38°C (excluding postoperative day 1), not responding to 48 hrs of antibiotics, and increased pain and tenderness in abdomen. If collection was found therapeutic aspiration of collection was done, the quantity and type (pus/bile/blood) of content were noted, and the fluid was sent for culture and sensitivity.

Postoperative superficial or deep incisional soft tissue SSIs and intra-abdominal abscess (organ/space SSI) were assessed by CDC defined criteria [2].

Statistical analysis of the data was carried out using SPSS 12.0 (SPSS, Inc., Chicago, IL). Categorical variables were analysed using a chi square test. Descriptive statistics for continuous variables were calculated by Student's *t*-test and the mean standard deviation was also expressed. A *p* value of < 0.05 was taken as statistically significant for all tests.

## 4. Results

Mean age of the patients was 37.70 ± 10.26 years. A total of 7 out of 113 patients, that is, 6% patients, developed SSIs. Most cases of SSI belonged to age group > 50 years, that is, 57% of overall SSIs. The mean age of patients developing SSI was 45.57 ± 8.89 years.

Female : male ratio in our study was 6.57 : 1. Gender was not associated with occurrence of SSI.

All the infections noted in our study were in the umbilical port site. There was no deep infection in any of our patients. Organism profile of the culture from SSIs showed monomicrobial infection: 58%* Staphylococcus aureus*, 14%* Pseudomonas*, 14%* E. coli*, and the rest of contaminants. Bacteriobilia was present in 32 patients out of 113 [28%], but only 5 patients developed SSIs amongst these 32 patients. The occurrence of SSIs in these patients with bacteriobilia was 16% as compared to 2% in patients without bacteriobilia. Fisher's exact test was used to find the statistical significance of this data. The *p* value was 0.018, which is statistically significant.

All the bile culture reports showed monomicrobial infections. Organisms found in culture of the patients with bacteriobilia were* E. coli* (75%),* Klebsiella* (19%),* Pseudomonas* (3%), and gram negative rods (3% species not specified).

A total of 18 patients had gallbladder content spillage and of these 18 only 1 patient developed SSIs; that is, these spillages were associated with surgical site infection in only 14% of patients. Statistically no significant difference in occurrence of SSIs in patients with gallbladder content spillage was found (see Tables 1 and 2).

## 5. Discussion

In recent years laparoscopy has become a preferential technique for cholecystectomy. It is performed more often than the classic surgery in most hospitals. SSIs are considered to be one of the most important surgical complications after any operative procedure. As per the study conducted by Jawein et al. the laparoscopy-attributable mortality reaches 0.5%, morbidity reaches 4%, and SSIs following this procedure range between 0.1 and 2% [3]. Laparoscopic surgeries in general are associated with a lesser number of infectious complications than open surgeries. In our study we evaluated the bile cultures and gallbladder content spillage as a possible cause for SSIs in LC. The SSIs rate in our study was around 6% of patients which was similar to SSIs rate noted by Den Hoed et al. (5.3%) [4]. Rate of wound infections varies greatly from 1.08% to 14.5% in the studies conducted by Jawien et al. and Malatani et al., respectively [3, 5].

All the infections noted in our study were in the umbilical port site. Gaur and Pujahari concluded that the umbilicus is the commonest site for sepsis following laparoscopic cholecystectomy. This may be because the deep umbilical depression is sometimes difficult to clean or it may be due to the routine protocol of our unit to extract the gallbladder through the umbilical port [6]. In our study we encountered only the superficial SSIs. None of the patients had deep or organ space infection, whereas Jawein noted superficial infections in 60.6%, deep infections in 21.2%, and organ/space SSIs in 18.2% [2]. Chang and Koc et al. found that the overall rate of infection was 1.1% and 2.1%, respectively [7, 8].

Various factors associated with SSIs in LC need to be evaluated further and definitive measures taken to decrease the morbidity associated with this procedure.

Female : male ratio in our study is 6.57 : 1, which is high compared to studies conducted by Assaff et al. and Suri et al., 1.32 : 1 and 3 : 1, respectively [9, 10]. This may correlate with the demographic variation. In our study none of the male patients developed SSIs. Our data however did not reveal any significant association between the gender and SSIs. Similarly, Chang et al. and Kumar et al. also did not find any statically significant association between the SSI and gender of the patient [7, 11].

Most of our patients belonged to the 3rd and 4th decades of life similar to study conducted by Suri et al. [10]. In our study a statistically significant association is found between the surgical site infection and age. SSIs are more common in old age patients probably due to lower immune response.

16% of patients had gallbladder content spillage in our study. This is low in comparison to the study conducted by Koc et al. and Assaff et al., that is, 34% and 41.3% of patients, respectively [8, 9]. No significant association was found between gallbladder content spillage and SSI. Suh et al. noted trocar site infections developed more frequently in patients with gallbladder perforation (*p* = 0.004) [12].

In our study the bile cultures were positive in 32 patients out of 113, that is, 28%. The rate of bile culture positivity was similar to other studies; that is, Malatani et al., Suri et al., and Mahafzah and Daradkeh also noted positive bile culture in 17.3%, 20%, and 27% of patients, respectively [5, 10, 13].

In our study we found that the occurrence of SSIs in patients with bacteriobilia was nearly 8 times more than in patients without bacteriobilia; also we found statistically significant association between bacteriobilia and SSIs. This may possibly be due to translocation of bacteria during retraction of the rectus and extraction of the gallbladder. But the organism profile in the bile and surgical sites are different. However, Malatani et al. and Mahafzah and Daradkeh noted no significant difference in the postoperative complication between the patients with a positive bile culture or sterile culture [5, 13]. Our organism profile in bile culture is similar to other studies such as Suri et al. and Valceanu et al.,* E. coli* being the commonest organism [10, 14]. Microorganism profile of the various SSIs depends on various factors including the type of surgery. In our study* Staphylococcus aureus* was the most common organism (58%). These results go in line with that of Cantlon et al., who found that* Staphylococcus aureus* was the most common pathogen followed by Enterobacteriaceae that were the second most frequently isolated organisms [15]. Also Kasatpibal et al. and Khorvash et al. recorded that* Staphylococcus aureus* had most frequency and the most common gram negative organisms were* Klebsiella*,* E. coli*, and* Pseudomonas* [16, 17].

## 6. Conclusion

Gallbladder content spillage does not lead to increase in SSIs. The occurrence of SSIs is significantly higher in patients with bacteriobilia. The risk of SSIs increases with age. Antibiotics in case of spillage should be offered only to elderly patients.

## Figures and Tables

**Table 1:** Distribution of SSIs in patients with gallbladder rupture.

Gallbladder content spillage	SSI present		SSI absent		Total	*p* value
	Number	Percentage	Number	Percentage	Number	
Present	1	14.28	17	16.03	18	1.000
Absent	6	85.72	89	83.97	95	
Total	7		106		113	

**Table 2:** Distribution of SSIs with bacteriobilia.

Bacteriobilia	SSI present		SSI absent		Total	*p* value
	Number	Percentage	Number	Percentage	Number	
Positive	5	4.42	27	23.9	32	0.0189
Negative	2	1.77	79	69.91	81	
Total	7	6.18	106	93.80	113	
